# Epidemiology and burden of alopecia areata in Taiwan: a systematic review

**DOI:** 10.3389/fmed.2025.1723424

**Published:** 2026-01-12

**Authors:** Chao-Chun Yang, Sheng-Hsiang Ma, Pei Jung Yen, Hung-Wei Lin, Kuei-An Chen, Chih-Chiang Chen

**Affiliations:** 1 Department of Dermatology, National Cheng Kung University Hospital, College of Medicine, National Cheng Kung University, Tainan, Taiwan; 2 International Center for Wound Repair and Regeneration, National Cheng Kung University, Tainan, Taiwan; 3 Department of Dermatology, Taipei Veterans General Hospital, Taipei, Taiwan; 4 Faculty of Medicine, School of Medicine, National Yang Ming Chiao Tung University, Taipei, Taiwan; 5 Institute of Public Health and Department of Public Health, National Yang Ming Chiao Tung University, Taipei, Taiwan; 6 Pfizer Ltd, Taipei, Taiwan; 7 Real World Solutions, IQVIA Solutions Taiwan, Taipei, Taiwan; 8 Department of Dermatology, National Yang Ming Chiao Tung University, Taipei, Taiwan; 9 Institute of Clinical Medicine, National Yang Ming Chiao Tung University, Taipei, Taiwan

**Keywords:** alopecia areata, comorbidities, epidemology, risk factors, Taiwan

## Abstract

**Introduction:**

Alopecia areata (AA) is a common autoimmune disorder characterized by nonscarring hair loss on the scalp and/or body, affecting individuals of all genders, ages, and races. AA can occur alongside various autoimmune, atopic, and psychiatric conditions, affecting patients’ quality of life. Although AA is a relatively common condition, recent epidemiological data regarding this condition in Taiwan are lacking.

**Methods:**

This systematic literature review aimed to assess the epidemiology, risk factors, and comorbidities associated with AA in Taiwan by analyzing articles published from January 2010 to June 2024.

**Results:**

A total of 37 studies were included, with most using the National Health Insurance Research Database (NHIRD) as the data source. The annual incidence and prevalence of AA in Taiwan were estimated at 0.011 and 0.014–0.016%, respectively. The mean age of AA onset ranged from 32 to 41.2 years, with a slight male predominance. Identified risk factors included autoimmune diseases, psychiatric disorders, viral infections, and lifestyle habits, while associated comorbidities involved chronic inflammatory dermatoses, atopic diseases, autoimmune diseases and mental disorders.

**Discussion:**

The review also highlighted evidence gaps, such as the need for validated diagnostic criteria in NHIRD and more research on ocular and systemic comorbidities. Given the significant disease burden of AA, further studies are needed to improve understanding and inform patient care and treatment strategies.

## 1 Introduction

Alopecia areata (AA) is one of the most prevalent dermatological diseases and is characterized by autoimmune, inflammatory, nonscarring hair loss on the scalp and/or body (1), affecting individuals of all genders, ages, and races. AA can coexist with other clinical conditions such as atopic dermatitis, thyroid disease, systemic lupus erythematosus (SLE), and other autoimmune diseases (2). Additionally, the clinical manifestations of AA may remain limited to either single or multiple patches with well-defined borders (localized AA) which may progress to complete scalp hair loss (alopecia totalis) or to total body hair loss (alopecia universalis) (2–5). AA also has a substantial psychological impact on patients in terms of anxiety and depression, and patients with AA also experience a decrease in quality of life in multiple domains, including health-related, social, and emotional (2, 4, 6, 7).

Epidemiological data are crucial for evaluating the overall disease burden and helping effective disease management. Although AA is a relatively common condition, there is a paucity of robust and recent epidemiological data (8). The prevalence of AA is 1 in 1000, with a lifetime incidence of about 2% worldwide (9). One recent study found that the total number of patients with AA in Taiwan increased over the years, highlighting the growing disease burden and potential unmet needs for AA patients in Taiwan (10). Racial differences regarding the epidemiology of AA have been reported in previous studies, showing that the Asian population has a higher prevalence of AA compared to individuals with a white ethnicity (8, 11, 12). It is therefore imperative to review currently published data from Taiwan to improve our understanding of the characteristics and disease burden of patients with AA.

This systematic review was carried out to (1) assess the epidemiology data on the prevalence and incidence of AA in Taiwan based on articles published between January 2010 and June 2024, and (2) investigate the disease burden of AA in Taiwan by identifying AA risk factors and comorbidities.

## 2 Methods

### 2.1 Search strategy

Literature screening following PRISMA guidelines was initiated for articles in PubMed and Cochrane databases published between January 1st, 2010 and June 14th, 2024 to identify relevant studies on AA from Taiwan. The PRISMA checklist is provided in Supplementary Material 1. A comprehensive search string was used by combining different terms to search across both databases.

### 2.2 Inclusion and exclusion criteria

Included studies were defined according to the Population, Intervention, Comparator(s), Outcomes, and Study design (PICOS) strategy (Supplementary Table S1). Case reports, review articles, and in-vitro studies were excluded. Review articles were excluded since the results may include findings from non-Taiwanese populations. Publications were excluded if PICOS criteria were not matched. The search strings used for the study were summarized in Supplementary Table S2.

**Figure 1:**
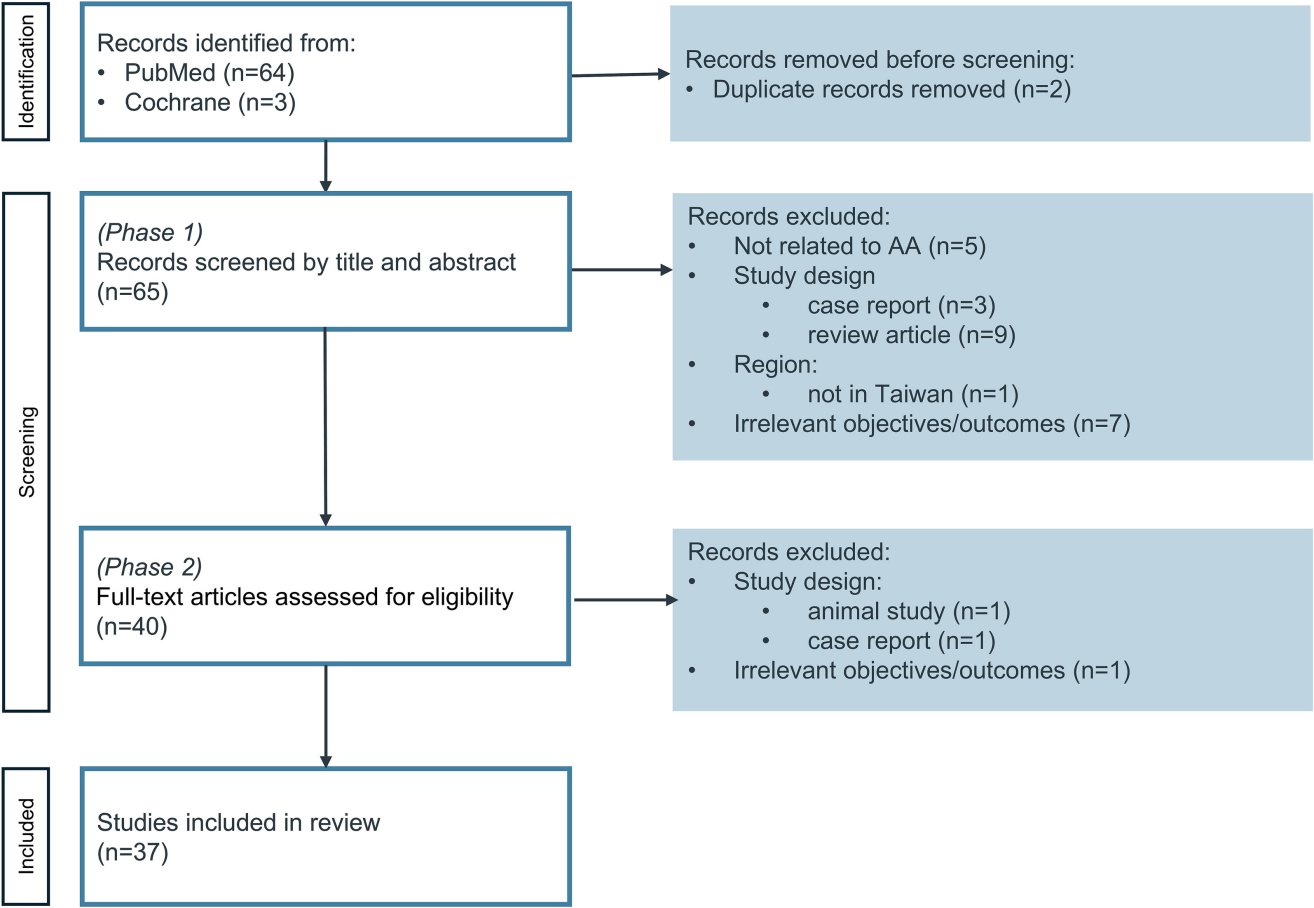
Diagram of study selection.

Two independent reviewers (Lin and Chen) were involved in a two-phase literature screening. In Phase 1, titles and abstracts were reviewed. Full-text manuscripts retained after Phase 1 were further reviewed in Phase 2. Following initial searches, publications were combined, and duplicates were removed (Figure 1). If two reviewers did not agree on inclusion, the third reviewer (Shau) was involved to reach a consensus.

Relevant articles on AA were screened, and studies reporting epidemiology, patient characteristics or risk factors were included. Articles in languages other than English were excluded. The validated Joanna Briggs Institute (JBI) critical appraisal checklists corresponding to the respective study designs were used by the reviewers to assess quality, and consensus was reached on the risk-of-bias rating. Detailed information is summarized in Supplementary Table S3.

The following information was extracted from the included articles: reference details, type of study, study period, number of cases, sample size, sex distribution, diagnosis criteria, severity classification criteria, mean/median age, age of onset, severity, prevalence/incidence, comorbidities, and risk factors.

## 3 Results

### 3.1 Search results

A total of 67 publications were identified (64 from PubMed and 3 from Cochrane). After applying the inclusion and exclusion criteria during the two-phase review, 37 studies were included for full-text review (Figure 1). Studies included in the review demonstrated acceptable methodological quality (Supplementary Table S3). All studies were considered to have a low (31 studies, 83.8%) to moderate (6 studies, 16.2%) risk of bias. Although many of the cohort studies lacked information on completion of follow-up (i.e., Q9 and Q10 of the JBI checklist), bias was considered minimal given the nature of the data source (i.e., NHIRD), as approximately 99% of the population was covered by Taiwan’s National Health Insurance system and had longitudinal follow-up until death or the end of the data period.

Publications were further classified into four categories: four publications reported the epidemiology, patient characteristics and/or treatment patterns of AA in Taiwan; 17 studies assessed the risk factors for developing AA; 10 studies investigated the comorbidities of patients with AA in Taiwan; and six studies performed a bi-directional analysis, which investigated both AA risk factors and comorbidities of AA in one study. (Table 1).

**Table 1:** Summary of selected studies.

Study number	References and year	Data source^†^	Study design	Data period^‡^	Study population	Outcomes of interest
1. Epidemiology, patient characteristics and/or treatment patterns of AA in Taiwan						
(24)	Wu 2013	Medical records from 2 sites in Taiwan	Longitudinal analysis	1987–2010	Late-onset AA (onset age at 50 years or above)	Patient characteristics and disease severity using guideline from US National Alopecia Areata Foundation (1999)
(27)	Weng 2016	NHIRD	Longitudinal analysis	2000–2011	Psoriasis with/without comorbidities (including AA)	Traditional Chinese Medicine Use
(26)	Wong 2022	Taiwan maternal and child health database	Longitudinal analysis	2004–2017	AA	Heritability of alopecia areata
(10)	Tsai 2024	NHIRD	Analysis 1: cross-sectional analysis Analysis 2: Longitudinal analysis	2016–2021	AA (severe and mild/moderate AA based on a claims-based algorithm)^§^	Prevalence and incidence of AA, demographics and treatment patterns
3. Comorbidities associated with AA patients						
(28)	Tsai 2011	NHIRD	Cohort study	2005–2008	Psoriasis	Comorbidities including AA
(30)	Chung 2015	NHIRD	Case–control study	1996–2011	Lichen planus patients	Autoimmune comorbid diseases including AA
(31)	Chen 2015	NHIRD	Cohort study	1996–2011	Vitiligo	Comorbidities including AA
(69)	Chiu 2017	NHIRD	Case control study	1997–2010	Sarcoidosis	Autoimmune diseases including AA
(70)	Liu 2019	NHIRD	Cohort study	2006–2013	Prostate cancer patients with androgen deprivation therapy	Autoimmune comorbid diseases including AA
(34)	Dai 2020	NHIRD	Cohort study	2001–2011	Major depressive disorder	Autoimmune skin diseases including AA
(41)	Dai 2020	National health interview survey + NHIRD	Cohort study	2001, 2005, 2009, 2013 NHIRD: until 2017	Smoker (current, former, never) Alcohol user (regular, social, never)	AA
(32)	Chang 2020	NHIRD	Cohort study	2000–2012	Rheumatoid arthritis	Alopecia
(42)	Li 2020	NHIRD	Nested Case–control study	1998–2013	Proton pump inhibitors users	AA
(18)	Ma 2021	NHIRD	Cohort study	1998–2013	Hepatitis C virus infection	Chronic inflammatory skin disease
(29)	Tu 2021	NHIRD	Cohort study	1997–2013	Human papillomavirus symptomatic infection	AA
(36)	Ho 2021	Taiwan Maternal and Child Health Database	Cohort study	2004–2017	Attention-Deficit/Hyperactivity Disorder with or without Methylphenidate Use	AA
(40)	Chang 2021	NHIRD	Cohort study	1998–2013	Polycystic ovary syndrome	AA
(37)	Dai 2021	NHIRD	Cohort study	2001–2011	Posttraumatic stress disorder	Autoimmune skin diseases including AA
(33)	Hsieh 2022	NHIRD	Cohort study	1999–2013	Ankylosing spondylitis	AA^¶^
(38)	Chou 2022	NHIRD	Cohort study	2001–2011	Obsessive-compulsive disorder	AA
(43)	Wang 2023	Taiwan/China Severe cutaneous adverse reaction consortium	Case–control study	2021–2022	COVID-19 vaccinations (27 new onset AA patients after COVID-19 vaccinations and 106 vaccines tolerant individuals)	Clinical characteristics and immune profiles of Immune-mediated alopecia
3. Assessment of risk factors of developing AA						
(15)	Chu 2011	NHIRD	Cohort study	1996–2008	AA	Comorbidity profiles
(16)	Chu 2012	NHIRD	Case–Control study	2000–2009	AA	Psychiatric comorbidities
(17)	Kang 2015	NHIRD	Cohort study	2004–2011 (patient identification)	AA	Stroke
(71)	Chen 2015	NHIRD	Cohort study	2001–2012 (patient identification)	AA	Herpes zoster
(14)	Chen 2016	NHIRD	Cohort study	2003–2009 (patient identification)	AA	Autoimmune diseases
(13)	Chen 2018	NHIRD	Cohort study	1997–2013	AA	Cancer
(18)	Ma 2020	NHIRD	Cohort study	1998–2011 (patient identification)	AA	Hearing loss
(25)	Li 2021	NHIRD	Cohort study	1998–2013	AA	Dementia
(44)	Ting 2022	NHIRD	Cohort study	1997–2013	AA	Retinal diseases
(72)	Wang 2023	NHIRD	Cohort study	1997–2013	AA	Suicide Attempt
4. Bi-directional analysis of risk factors/comorbidities						
(19)	Wei 2020	NHIRD	Cohort study	1998–2011	Bidirectional association between AA and atopic dermatitis	
(35)	Dai 2020	NHIRD	Cohort study	1996–2011	Bidirectional association between AA and major depressive disorder (proband and unaffected siblings, born before 1990)	
(22)	Dai 2020	NHIRD	Cohort study	Not mentioned; follow up until end of 2011	Bidirectional association between AA and sleep disorders	
(23)	Dai 2021	NHIRD	Cohort study	1998–2011 (patient identification period)	Bidirectional association between AA and migraine	
(21)	Dai 2022	NHIRD	Cohort study	1998–2013	Bidirectional association between AA and irritable bowel syndrome	
(20)	Dai 2021	NHIRD	Cohort study	1998–2011 (patient identification period)	Bidirectional association between AA and thyroid diseases	

AA, alopecia areata; NHIRD, National Health Insurance Research Database.

^†^NHIRD includes full population dataset or sampling dataset (longitudinal health insurance database, LHID).

^‡^Data period includes baseline, patient identification and follow up period if not indicated.

^§^Severe patients included 90 days of systemic steroid used for AA, topical intralesional injectable steroids (>9 cm^2^), 90 days immunosuppressants use for AA, at least one record of topical immunotherapy treatment or use of phototherapy for AA.

^¶^Alopecia (ICD-9-CM = 740.0x) and its subtypes (androgenetic alopecia, ICD-9-CM: 704.00 and 704.09 and alopecia areata, ICD-9-CM: 704.01 and 704.02) were investigated in the study. The outcomes related to alopecia areata are reported in the current study.

Among the 37 included publications, the National Health Insurance Research Database (NHIRD) was the most commonly used data source (*n* = 33). NHIRD is a claims database which covers 99% of the population in Taiwan.

### 3.2 Incidence and prevalence of AA in Taiwan

In the latest study using the full-population NHIRD in Taiwan, the total number of patients with AA in Taiwan increased from 3,221 in 2016 to 3,855 in 2020 (with a prevalence of 0.14–0.16 per 1,000), with about 8% of patients having severe AA and most were newly diagnosed. During 2017–2020, there was an average of 2,659 incident patients with AA in Taiwan annually, with a crude incidence rate of about 0.11 per 1,000 (10). In earlier studies using the data from 1997 to 2011, the estimated prevalence were ranged from 4.1 to 7.1 per 1,000 and the estimated incidence rate were 0.36–1.01 per 1,000 (13–19).

### 3.3 Characteristics of patients with AA in Taiwan

A summary of studies that identified AA cohorts is presented in Table 2. In the study cohort identified from the NHIRD before 2013, the mean age of initial AA diagnosis was around 32 years, and the proportion of male patients was around 50% (13, 15–17, 19–23). In a recent cohort (2017–2018) identified by Tsai et al. (10), the mean age of patients with AA was 41.2 years, with a slight male predominance (male: 53.4%; female: 46.6%). In contrast, there was a female predominance in cohorts with older age: 67% of patients were female in a late-onset AA cohort (patients with first onset of AA at 50 years or above) (24). A similar distribution was found in an AA cohort aged 45 years or above (25).

**Table 2:** Summary of AA cohorts identified in the publications.

Study no. and references	Study type	Data source	Cohort identification period	Definition of AA^†^	AA cohort (*n*)^‡^	Age at first diagnosis (years)	Sex (% of male)
(15)	Cohort study	1 Mn NHIRD sample	1996–2008	ICD-9-CM: 704.01 by dermatologist	4,334 I: 0.43; P:4.33	Mean (SD): 32.2 (14.8)	49%
(16)	Cohort study	1 Mn NHIRD sample	2000–2009	ICD-9-CM: 704.01 outpatient visit by dermatologist	5,117 P: 4.33	Median: 31	49%
(17)	Cohort study	1 Mn NHIRD sample	2004–2011	ICD-9-CM: 704.01 during ambulatory care visit	4,065 (all AA) I: 0.51; P. 4.07 3,231 (after excluded history of stroke and age <18)	Mean: 36.1	49.2%
(14)	Cohort study	1 Mn NHIRD sample	2003–2009	ICD-9-CM: 704.01 during ambulatory care visit	4,665 (all AA) I: 0.67; P: 4.67 3448 (After excluded history of autoimmune diseases and age <18)	NR	NR
(13)	Cohort study	NHIRD^§^	1997–2003	Primary diagnosis of ICD-9-CM: 704.01 by dermatologists Without previously cancer	162,499 I: 1.01; P: 7.07	Mean (SD): 32.3 (14.8) Median:30.9	47.97%
(18)	Cohort study	NHIRD^§^	1998–2011	ICD-9-CM: 704.01 by dermatologists	5,002 I: 0.36; P: 5.00	Mean (SD): 37.4 (12.2)	49.1%
(19)	Cohort study; Bi-directional analysis – AA cohort	3 Mn NHIRD sample	1998–2008	ICD-9-CM: 704.01 ≥3 visits by dermatologists or rheumatologists	13,931 (before matching) I: 0.42; P: 4.31	Median: 30.8 (12,022 after matching)	48.9% (12,022 after matching)
(35)	Cohort study; Bi-directional analysis – AA proband cohort	1 Mn NHIRD sample	1996–2011	ICD-9-CM: 704.01 ≥3 visits by dermatologists Born before 1990	2,123	Median: 31.3	44.8%
(22)	Cohort study; Bi-directional analysis – AA cohort	NHIRD^§^	Not mentioned; follow up until end of 2011	ICD-9-CM: 704.01 ≥3 visits by dermatologists ≥20 years Without previous sleep disorders	5,648	Median: 34.1	52.2%
(25)	Cohort study	NHIRD^§^	1998–2011	ICD-9-CM: 704.01 ≥2 visits by dermatologists Age ≥45 Without previous dementia	2,534	Mean (SD): 53.9 (7.5)	42.4%
(23)	Cohort study; Bi-directional analysis – AA cohort	NHIRD^§^	1998–2011	ICD-9-CM: 704.01 by dermatologist	5,608	Median: 32.7	50.1%
(20)	Cohort study; Bi-directional analysis – AA cohort	NHIRD^§^	1998–2011	ICD-9-CM: 704.01 ≥3 visits by dermatologists Excluded patients with thyroid diseases	5,929	Median: 32.6	51.9%
(44)	Cohort study	NHIRD^§^	1997–2012	ICD-9-CM: 704.01 ≥3 visits by dermatologists ≥3 years	9,909	Median: 31.6	48.5%
(21)	Cohort study; Bi-directional analysis – AA cohort	NHIRD^§^	1998–2011	ICD-9-CM: 704.01 ≥3 visits by dermatologists Excluded previous IBS	5,446	Mean (SD): 34.1 (13.5)	49.5%
(72)	Cohort study	NHIRD Sampling database	1997–2013	ICD-9-CM: 704.01 ≥3 outpatient or 1 inpatient visit by dermatologists	10,515 (only ≥10 y)	Median: 33	48.8%
(10)	Cohort study (Longitudinal analysis)	NHIRD full population dataset	2017–2018	ICD-10-CM: L63 ≥3 visits by dermatologists or rheumatologists With at least 90 days between first and last claim Without other hair loss disorders^¶^	Before matching: 6,016 Severe: 477 Mild/moderate: 5,539	Mean (SD):41.2 (14.17)^††^ Median: 41^††^	53.4%

I, estimated incidence per 1,000; ICD-9-CM, International Classification of Disease – 9th version- clinical modification; ICD-10-CM, International Classification of Disease – 10th version- clinical modification; Mn, million; NHIRD, National Health Insurance Research Database; P, estimated prevalence per 1,000.

^†^ICD-9-CM: 704.01 (Alopecia areata); ICD-10-CM: L63 (Alopecia areata).

^‡^Unmatched cohort if not indicated. The incidence and prevalence were estimated based on the population included in the study and number of AA identified.

^§^Not addressed in the study whether the full population dataset of sampling database (Longitudinal Health Insurance Database, LHID) was used.

^¶^Hair loss included trichotillomania (F63.3), telogen effluvium (L65.0), tinea capitis and tinea barbae (B35.0), scarring alopecia (L66.1, L66.3, L66.4, L66.8, L66.9), unspecified nonscarring hair loss (L65.9), pseudopelade (L66.0), folliculitis decalvans (L66.2), and other specified hair loss (L65.1, L65.2, L65.8).

^††^Defined as the first occurrence of AA health claim during the index period (2017–2018).

The heritability of AA has also been observed in the Taiwanese population. A study using the Taiwan Maternal and Child Health Database found that children of parents with AA have twice the risk of developing AA compared to offspring of parents who did not have the condition (26).

### 3.4 Treatment patterns of AA in Taiwan

Topical corticosteroids were the most common treatment for patients with AA (80% of patients) (10). The treatment patterns varied depending on disease severity, with 48.5 and 12.0% of patients with severe AA receiving systemic glucocorticoids and immunosuppressants, respectively, compared to only 15.7 and 0.7% of patients with mild or moderate AA (10). Traditional Chinese Medicine (TCM) was noted as a potential treatment option in patient with AA. A study assessing the psoriasis treatment patterns found that patients with psoriasis preferred to visit TCM practitioners when they had several comorbidities, including AA (adjusted prevalence rate ratio: 1.36 [95% confidence interval (CI): 1.06–1.75]) (27).

### 3.5 Risk factors for developing AA in the Taiwanese population (Table 3)

**Table 3:** Risk factors of developing AA in Taiwanese population.

Risk factors	AA cases (*n*)/exposure (*N*)	AA cases (*n*)/control (*N*)	Relative risk estimation	Results (95% CI)	References
Chronic inflammatory dermatoses					
Psoriasis	43/51,800	44/207,200	aRR	4.71 (2.98–7.45)	Tsai (28)
Psoriasis	7/5,179	194/320,415	aHR	2.44 (1.14–5.21)	Tu (29)
Lichen planus	126/12,427	145/49,708	aOR	2.82 (2.20–3.62)	Chung (30)
Vitiligo	270/14,883	179/59,532	aOR	5.11 (4.20–6.21)	Chen (31)
Atopic dermatitis	327/40,307	214/161,228	aHR	6.00 (5.04–7.14)	Wei (19)
Autoimmune diseases					
Rheumatoid arthritis	37/22,276	16/25,792	aHR	2.64 (1.47–4.76)	Chang (32)
Ankylosing spondylitis	5/28,825^††^	20/113,637^††^	aHR	0.98 (0.37–2.62)	Hsieh (33)
Mental disorders					
All mental disorders^†^	48/67,921^††^	153/257,673^††^	aHR	1.47 (1.05–2.06)	Tu (29)
Major depressive disorder	694/222,522	213/890,088	aHR	11.61 (9.92–13.59)	Dai (34)
Major depressive disorder	75/16,543	160/69,408	aHR	1.66 (1.24–2.22)	Dai (35)
Unaffected siblings of MDD patients	65/17,352	160/69,408	aHR	1.64 (1.27–2.12)	
Attention-deficit/hyperactivity disorder	88/90,016	1191/1,660,440	aHR	1.30 (1.04–1.64)	Ho (36)
Posttraumatic stress disorder	24/10,967	30/43,868	aHR	4.77 (2.47–9.20)	Dai (37)
Obsessive-compulsive disorder	154/44,324	41/177,296	aHR	13.69 (9.38–19.98)	Chou (38)
Infectious diseases					
hepatitis C virus infection	NR	NR	aHR	6.69 (4.28–10.44)	Ma (39)
Human papillomavirus infections	57/30,001	144/30,001	aHR	2.55 (1.88–3.47)	Tu (29)
Thyroid diseases					
Rheumatoid arthritis patients with thyroid diseases^‡^	5/1,764	NR	aHR	3.53 (1.38–9.05)	Chang (32)
Thyrotoxicosis	107/35,071	116/350,710	aHR	9.29 (7.11–12.14)	Dai (20)
Graves’ disease	57/19,227	66/192,270	aHR	8.66 (6.03–12.42)	
Thyroiditis	13/5,460	20/54,600	aHR	6.42 (3.15–13.11)	
Hashimoto thyroiditis	3/3,352	12/33,520	aHR	2.70 (0.75–9.70)	
Other diseases					
Migraine	29/16,650	37/66,600	aHR	1.96 (1.15–3.32)	Dai (23)
Polycystic ovary syndrome	24/10,967	30/43,868	aHR	3.12 (1.81–5.40)	Chang (40)
Combined sleep disorders	308/93,130	NR/372,520	aHR	4.70 (3.99–5.54)	Dai (22)
Obstructive sleep apnea	20/93,130	NR/372,520	aHR	3.89 (2.46–6.16)	
Non-apnea insomnia	288/93,130	NR/372,520	aHR	4.77 (4.03–5.64)	
Irritable bowel syndrome	116/56,429	86/255,716	aHR	5.38 (3.95–7.34)	Dai (21)
Other risk factors					
Smoking (current smoker)^§^	42/12,964	106/44,275	aHR	1.88 (1.22–2.88)	Dai (41)
Smoking (former smoker)	6/2,816	106/44,275	aHR	1.61 (0.68–3.83)	
Alcohol (social)	34/15,611	108/36,888	aHR	0.65 (0.43–0.98)	
Alcohol (regular)	12/7,556	108/36,888	aHR	0.49 (0.26–0.93)	
PPI (cDDD = 31–120)^¶^	7,561^‡‡^	7,605^§§^	aOR	1.12 (1.06–1.18)	Li (42)
PPI (cDDD = 121–365)^¶^	4,651^‡‡^	4,914^§§^	aOR	1.22 (1.15–1.29)	
PPI (cDDD > 365)^¶^	1,160^‡‡^	948^§§^	aOR	1.40 (1.27–1.54)	

AA, alopecia areata; aHR, adjusted hazard ratio; aRR, adjusted relative risk; aOR, adjusted odds ratio; cDDD, cumulative defined daily dose; CI, confidence interval; MDD, major depressive disorder NR, not reported; PPI, proton pump inhibitors.

^†^ICD-9-CM code of 292–302 were included.

^‡^The risk of patients’ Rheumatoid arthritis patients with thyroid diseases.

^§^Nonsmoker as control group.

^¶^cDDD ≤ 30 as the control group.

^††^events per person-year.

^‡‡^Li 2020 was a nested case–control study, 17,776 AA patients were identified as “case” group: 7,561 were cDDD = 31–120; 4,651 were cDDD = 121–365 and 1,160 were cDDD > 365.

^§§^17,776 control (without AA) group was also identified: 7,605 were cDDD = 31–120; 4,914 were cDDD = 121–365 and 948 were cDDD > 365.

Sixteen studies assessed diseases which could be associated with a higher risk of developing AA. Autoimmune/atopic diseases and psychiatric disorders were the two major disease areas studied.

Patients with chronic inflammatory dermatoses, such as atopic dermatitis (19), psoriasis (28, 29), lichen planus (30), and vitiligo (31) were found to be associated with a higher risk of developing AA. Rheumatoid arthritis also increased the risk of AA (32), but not ankylosing spondylitis. (33)

The association between mental disorders and the risk of AA was also widely investigated. Patients with mental disorders, including major depressive disorder (MDD) (34, 35), attention-deficit/hyperactivity disorder (ADHD) (36), posttraumatic stress disorder (37), and obsessive-compulsive disorder (38) were found to have an increased risk of AA. The study by Dai et al. (35) further investigated the relationship between MDD family history and AA by linking the NHIRD data and birth certificates. The results showed that both MDD probands and their unaffected siblings had a higher risk of AA compared to the control group.

Other identified risk factors for AA include viral infections (Hepatitis C virus and Human papillomavirus) (29, 39), irritable bowel syndrome (21), sleep disorders (22), thyroid disorders (including thyrotoxicosis, Graves’ disease, and thyroiditis) (20, 32), migraine (23), and polycystic ovary syndrome (40).

The association between lifestyle habits or medication use and the risk of AA was studied in the Taiwanese population. Smokers (current of former) were found to have a higher risk of developing AA from a study using National Health Interview Survey database (conducted in 2001, 2005, 2009, and 2013) and the NHIRD (41). Exposure to proton pump inhibitors was found to increase the risk of AA. Compared to a cumulative defined daily dose (cDDD) ≤ 30, patients with a higher cDDD had a higher risk of AA, and a dose-dependent trend was also observed (42). In addition, among patients with ADHD, the use of methylphenidate did not increase the risk of AA compared to patients not using methylphenidate (36).

Risk factors for coronavirus disease 2019 (COVID-19) vaccine-related AA were investigated using Taiwan/China Severe Cutaneous Adverse Reaction consortium data, with 27 cases of COVID-19 vaccine-related AA and 106 vaccine-tolerant controls included. Underlying chronic urticaria and nail dystrophy were found to be associated with COVID-19 vaccine-related AA. Additionally, laboratory findings such as increased antinuclear antibody level, total IgE level, and eosinophil count were also risk factors for developing COVID-19 vaccine-related AA (43).

### 3.6 AA-related comorbidities in the Taiwanese population

Studies investigating AA-related comorbidities are summarized in Table 4. Tsai et al. (10) identified a study cohort of 6,016 patients with AA in Taiwan during 2017–2018, with 477 cases of severe AA and 5,539 cases of mild/moderate AA. Baseline comorbidities of AA (i.e., at first diagnosis of AA) were analyzed. Allergic rhinitis (6.13%) and allergic conjunctivitis (3.21%) were the two major coexisting atopic conditions at baseline. Within conditions included in the Charlson Comorbidities Index, diabetes (with or without complications, 4.54%), peptic ulcer (3.27%), and mild liver disease (3.27%) were the three main comorbidities at baseline. The prevalence of comorbidities was similar between patients with severe or mild/moderate AA except for allergic urticaria (2.31% in severe AA and 0.54% in mild/moderate AA).

**Table 4:** Comorbidities associated with AA.

Comorbidities	Cases (*n*)/AA cohort (*N*)	Cases (*n*)/Control cohort (*N*)	Relative risk estimation	Results (95% CI)	References
Chronic inflammatory dermatoses					
Atopic dermatitis	487/12,022	355/48,088	aHR	5.47 (4.76–6.28)	Wei (19)
Atopic dermatitis	218/4,334	14654/784,158	aOR	2.24 (1.95–2.58)^‡^	Chu (15)
Psoriasis	13/3,448	35/17,240	aHR	2.02 (1.06–3.83)	Chen (14)
Psoriasis	81/4,334	5184/784,158	aOR	2.80 (2.24–3.50)^‡^	Chu (15)
Vitiligo	14/4,334	430/784,158	aOR	5.23 (3.06–9.00)^‡^	
Autoimmune diseases					
Autoimmune disease	52/3,448	137/17,240	aHR	1.86 (1.32–2.63)	Chen (14)
Rheumatoid arthritis	20/3,448	55/17,240	aHR	1.79 (1.07–3.00)	
Rheumatoid arthritis	45/4,334	6329/784,158	aOR	1.69 (1.26–2.28)^†^	Chu (15)
				1.27 (0.94–1.72)^‡^	
Lupus erythematosus	64/4,334	2468/784,158	aOR	3.95 (3.05–5.11)^‡^	
Systemic lupus erythematosus	10/3,448	10/17,240	aHR	5.01 (2.08–12.05)	Chen (14)
Atopic diseases					
Asthma	245/4,334	43983/784,158	aOR	1.24 (1.09–1.41)^†^	Chu (15)
			aOR	1.07 (0.93–1.22)^‡^	
Allergic rhinitis	618/4,334	87064/784,158	aOR	1.29 (1.18–1.41)^‡^	
Mental disorders					
Any psychiatric disease	412/5,117	1246/20,468	aOR	1.36 (1.21–1.53)	Chu (16)
Anxiety	257/5,117	672/20,468	aOR	1.52 (1.30–1.78)	
Attention deficit disorder	19/5,117	74/20,468	aOR	0.98 (0.59–1.63)	
Bipolar	29/5,117	103/20,468	aOR	0.91 (0.58–1.43)	
Depression	146/5,117	444/20,468	aOR	1.16 (0.94–1.42)	
Manic	11/5,117	32/20,468	aOR	1.36 (0.66–2.83)	
Obsessive-compulsive disorder	24/5,117	56/20,468	aOR	1.58 (0.96–2.60)	
Phobia	12/5,117	33/20,468	aOR	1.11 (0.56–2.18)	
Personality disorder	22/5,117	60/20,468	aOR	1.29 (0.78–2.13)	
Schizophrenia	35/5,117	227/20,468	aOR	0.54 (0.37–0.78)	
MDD in AA proband	167/2,123	94/9,192	aHR	8.22 (6.41–10.54)	Dai (35)
MDD in AA unaffected siblings	64/2,298	94/9,192	aHR	2.55 (1.91–3.40)	
Suicide Attempt	61/10,515	81/105,150	aHR	6.28 (4.47–8.81)	Wang (72)
Neurological diseases					
Hemorrhagic stroke	NR/3,231	NR/16,155	aHR	2.18 (1.01–4.84)	Kang (17)
Ischemic stroke	NR/3,231	NR/16,155	aHR	1.58 (1.00–2.45)	
Unspecified stroke	NR/3231	NR/16,155	aHR	2.27 (1.19–4.31)	
Any dementia	32/2,534	83/25,340	aHR	3.24 (2.14–4.90)	Li (25)
Alzheimer’s dementia	5/2,534	10/25,340	aHR	4.34 (1.45–12.97)	
Vascular dementia	4/2,534	14/25,340	aHR	2.05 (0.64–6.63)	
Other dementia	23/2,534	59/25,340	aHR	3.36 (2.06–5.48)	
Migraine	45/5,608	40/22,432	aHR	3.26 (2.12–5.01)	Dai (23)
Thyroid diseases					
Hashimoto’s thyroiditis	5/3,448	10/17,240	aHR	2.47 (0.84–7.26)	Chen (14)
Hashimoto’s thyroiditis	8/5,929	18/59,290	aHR	4.35 (1.88–10.04)	Dai (20)
Toxic nodular goiter	19/5,929	18/59,290	aHR	10.17 (5.32–19.44)	
Nontoxic nodular goiter	54/5,929	102/59,290	aHR	5.23 (3.76–7.28)	
Hyperthyroidism	89/5,929	112/59,290	aHR	7.96 (6.01–10.54)	
Graves’ disease	47/5,929	57/59,290	aHR	8.36 (5.66–12.35)	
Thyroiditis	13/5,929	32/59,290	aHR	4.04 (2.12–7.73)	
Cancer					
Overall cancer	2,099/162,499				Chen (13)
Nonhematologic cancer	1,993/162,499	NR	SIR^§^	1.10 (1.05–1.15)	
Upper GI cancer	136/162,499	NR	SIR^§^	0.70 (0.58–0.82)	
Liver cancer	192/162,499	NR	SIR^§^	0.82 (0.71–0.94)	
Nonmelanoma skin cancer	30/162,499	NR	SIR^§^	0.59 (0.38–0.80)	
Female breast cancer	395/162,499	NR	SIR^§^	2.93 (2.64–3.22)	
Uterine and cervix cancer	150/162,499	NR	SIR^§^	0.84 (0.70–0.97)	
Kidney and urinary bladder cancer	113/162,499	NR	SIR^§^	2.95 (2.41–3.50)	
Hematologic cancer	112/162,499	NR	SIR^§^	1.19 (0.97–1.41)	
Lymphoma cancer	75/162,499	NR	SIR^§^	1.55 (1.20–1.90)	
Other diseases					
Obstructive Sleep Apnea	45/5,648	NR/22,592	aHR	3.80 (2.53–5.71)	Dai (22)
Non-apnea insomnia	438/5,648	NR/22,592	aHR	4.20 (3.68–4.79)	
Retinal disease (Total)	61/9,909	175/99,090	aHR	3.10 (2.26–4.26)	Ting (44)
Retinal detachment	13/9,909	33/99,090	aHR	3.98 (2.00–7.95)	
Other retinopathy	42/9,909	108/99,090	aHR	3.24 (2.19–4.81)	
Retinal vascular occlusion	11/9,909	44/99,090	aHR	2.45 (1.22–4.92)	
Herpes Zoster	NR	NR	aOR	3.74 (3.28–4.27)	Chen (71)
Irritable bowel disease	128/5,446	90/21,784	aHR	5.20 (3.97–6.82)	Dai (21)
Hearing loss	33/5,002	75/50,020	aHR	4.18 (2.78–6.31)	Ma (18)

AA, alopecia areata; aHR, adjusted hazard ratio; aOR, adjusted odds ratio; CI, confidence interval; GI, gastrointestinal; MDD, major depressive disorder NR, not reported; SIR, standardized incidence ratio.

^†^Chu 2012 reported the results (adjusted odds ratio) by 2 models. Results from the model adjusted for age and sex.

^‡^Chu 2012 reported the results (adjusted odds ratio) by 2 models. Results from model adjusted for age, sex, and other comorbid diseases.

^§^SIR calculated by the number of cancer cases that arose among AA patients divided by the expected number of cancer cases according to national age- specific, gender- specific, and period- specific cancer rates. Yearly reports of cancer rates were obtained from Taiwan National Cancer Registry.

Similar to studies on risk factors of AA, publications investigating the association between comorbidities and AA mainly focused on chronic inflammatory dermatoses, atopic diseases, autoimmune diseases, and mental disorders. AA was associated with an increased risk of atopic dermatitis, allergic rhinitis, psoriasis, vitiligo, rheumatoid arthritis, and lupus erythematosus (14, 15, 19). However, the risk of asthma in patients with AA was not significantly different from that of patients without AA after adjusting for age, sex, and other comorbid diseases (15). Patients with AA were also found to have an increased risk of mental disorders and neurological diseases. The risk of any psychiatric diseases, anxiety, schizophrenia (16), major depression disorder (35), dementia, and stroke (including hemorrhagic and ischemic stroke) (17) was significantly higher in patients with AA than in the control group.

The risk of cancer in patients with AA was also assessed in several studies. The overall cancer risk in patients with AA was slightly lower, but there was a slightly higher incidence ratio of certain types of cancer in patients with AA compared to the control group (13). The risk of female breast cancer, kidney and urinary bladder cancer, and lymphoma were elevated in patients with AA, while the risk of nonmelanoma skin cancer, upper GI cancer, liver cancer, and uterine and cervix cancer were lower in patients with AA than in the general population (13).

In addition, patients with AA were also associated with the increased risk of having sensorineural hearing loss (18) and retinal detachment (44).

## 4 Discussion

To the best of our knowledge, this is the first systematic literature review study on AA epidemiology and disease burden in Taiwan. By reviewing 37 articles, we summarized the epidemiology of AA in Taiwan, risk factors for developing AA, and AA-related comorbidities. In addition, demographics of patients with AA in Taiwan could be understood through the analysis of newly diagnosed AA cohorts reported in studies.

Although the modelling study by Jeon et al. (45) reported that the prevalence estimates tend to be higher in Asian regions, the estimated annual incidence rate and prevalence of AA in Taiwan reported in the latest study (estimated by total population in each year in Taiwan during 2017–2020) were 0.011 and 0.015%, respectively (10), which are lower than data reported in other countries. For instance, the estimated global incidence of AA varies between 0.1 and 3.8%, and the prevalence of AA is 0.1% (9, 45–47). In other Asian countries, the incidence rate in South Korea was 0.2%; and the prevalence ranged from 0.16 to 0.19% in Japan and 0.37% in South Korea (46, 48). A UK study showed a threefold higher AA incidence in people of Asian origin compared to those of white ethnicity (8). The geographic region, social factors, lifestyle and could cause differences in prevalence and incidence rate of AA (45). In addition to these factors, the lower incidence and prevalence of AA in Taiwan might be due to stricter case definition used in the study by Tsai et al. (i.e., ≥3 claims with AA diagnosis by dermatologists or rheumatologists) compared to other studies that used a one-time diagnosis as the inclusion criterion (46, 48); AA patients without persistent medical consultation or treatment could therefore not be captured in the study. Moreover, although the ICD codes used for AA were inconsistent with the codes used in other studies (48, 49), underestimation of the number of patients with AA might exist: for example, a US study also included ICD-9-CM: 704.09 (Other alopecia) to define AA (50), and clinicians might code it with other hair loss disorders.

Chronic inflammatory dermatoses, autoimmune diseases, atopic diseases, and psychiatric diseases were major disease categories when assessing the disease burden of AA in Taiwan, as summarized in Tables 3, 4. We observed that Taiwanese patients with AA showed various systemic and psychiatric comorbidities. This observation aligns with findings from other countries, including Korea, UK, and the US (51–55).

The activation of T helper cell (Th) 2, Th1, Th17 and Th9 cytokines play a vital role in the disease pathogenesis of AA. Since AA shares overlapping immune-mediated and inflammatory pathways with other autoimmune and atopic disorders, such as mediation of T-cells or activation of cytokines (54, 56–58), autoimmune diseases were one of the comorbidities that had the strongest association with AA (54). Consistent with findings in Taiwan, observational studies from the UK reported an increased risk of autoimmune conditions among patients with AA (adjusted HR for combined autoimmune conditions: 1.45 [95% CI: 1.28–1.66] (51). Furthermore, a meta-analysis encompassing 102 studies and including 680,823 patients with AA and 72,011,041 controls demonstrated a significant association between AA and autoimmune diseases (meta-analyzed odds ratio 2.28 [95% CI 1.75–2.97]) (54). In contrast to other autoimmune diseases, a meta-analysis and an observational study reported that ankylosing spondylitis had no association with AA (33, 54) and concluded that no direct association between AA and ankylosing spondylitis can be made (33).

Stressful phenomena may lead to hair loss through immune dysregulation and may trigger the functional equivalent of the hypothalamic–pituitary–adrenal axis in hair follicles, resulting in neurogenic inflammation and inducing premature destruction of the follicle (55, 59–61). Consistent with the study by Chu et al. (16), Okhovat et al. (55) reported that the combined odds ratios of anxiety and depression were 2.5 (95% CI: 1.54–4.06) and 2.71 (95% CI: 1.52–4.82), respectively, in a meta-analysis. Moreover, studies in Taiwan found that patients with AA had a higher risk of developing mental disorders such as bipolar and manic disorders, while attention-deficit/hyperactivity disorder, post-traumatic stress disorder, and obsessive-compulsive disorder were risk factors for developing AA. Bi-directional associations between MDD and AA were both reported by Dai et al. (35) in a Taiwanese population and by Vallerand et al. in the United Kingdom (62).

In addition to autoimmune and psychiatric diseases, ocular disorders (fundus changes, disorders of the sclera, lens changes, inflammation of the eyelid, iridocyclitis, keratitis, lacrimal system disorders, disorders of the choroid or retina and dry eye disorder) were found to have a strong association with AA in previous studies (44, 63, 64). Several hypotheses have been proposed to support the underlying mechanisms linking AA and ocular involvement. T-cell-mediated autoimmune condition may play a major role in the pathogenesis of AA and ocular disease. One key feature of AA pathogenesis is the collapse of hair follicle immune privilege, which normally protects follicular structures from autoimmune attack. Similarly, the anterior chamber of the eye maintains an immune-privileged environment to prevent inflammatory damage. Disruption of this ocular immune privilege may trigger local immune responses, contributing to ocular pathology in patients with AA (64, 65). Based on the studies review, only retinal disease has studied in Taiwan by using claims data. Further research should explore a border range of ocular diseases, ideally the data sources contains results on clinical examinations (e.g., complete ophthalmologic examination involving detailed anterior segment and fundus examination) to better elucidate potential etiological mechanisms.

We identified key evidence gaps on AA in Taiwan. First, comprehensive epidemiology data on AA in Taiwan was limited, and validation of AA diagnosis in NHIRD is required. Even though AA cohorts were identified in 12 studies, as listed in Table 2, the heterogeneity of AA diagnostic algorithms makes the results from different studies, especially epidemiological data, difficult to compare. The AA cohorts were identified using the national claims in two different data periods: before 2015 (inclusive, ICD-9-CM era) and after 2016 (inclusive, ICD-10-CM era). Inconsistencies were found in several conditions (66), which highlights that the comparison between studies from two different periods should be discussed with caution. Given the published epidemiological data on AA in Taiwan differ from those reported in other countries, future research should focus on developing and validating the algorithm for defining AA cases in real-world data sources to ensure the accuracy and consistency.

Accurate diagnosis and grading of AA severity are essential for assessing the true disease burden (67, 68). However, only two of the included studies reported disease severity. Wu et al. (24) classified the severity of the AA as S1-S4 based on the guidelines from National Alopecia Areata Foundation. Although NHIRD lacks clinical information, the algorithm proposed by Tsai et al. could be a potential approach to identify moderate-to-severe AA by the presence of advanced treatment (immunosuppressants, topical immunotherapy treatment, topical intralesional injectable steroids, or phototherapy) (28). Further validation studies on the algorithm should be taken by linking the NHIRD to data sources with clinical information.

The studies included in this review shared similar potential biases, and the causality of comorbidities and risk factors could not be confirmed. To elaborate, 33 out of 37 included studies were from the same database (NHIRD), which shares the same limitations. Most epidemiologic and comorbidity analyses were based on overlapping time windows within the same national claims population, meaning these findings do not represent independent cohorts. Beyond autoimmune/atopic and mental disorders, many of the comorbidities/risk factors were reported in a single study. Therefore, randomized controlled studies are required to confirm the causality, and patient-reported outcomes can be assessed to address the burden on patients’ quality of life.

Lastly, several comorbidities have been reported to be associated with AA; however, they have not been well studied in the Taiwanese population. For example, a meta-analysis by Ly et al. (54) reported that patients with AA have a higher risk of developing cardiovascular diseases (pooled odds ratio: 1.31 [95% CI: 1.05–1.63]), particularly coronary artery disease and dyslipidemia. These findings highlight potential directions for future research.

Even though the review followed PRISMA guidelines and used both the term “alopecia areata” and its corresponding MeSH term to capture relevant studies, there remains a potential limitation: some studies indexed under broader hair-loss terms or alternative diagnostic wording may not have been captured. In summary, the current systematic literature review suggests that AA has an overall impact on patients further than an aesthetic concern, causing a significant disease burden and emphasizing the need for effective treatments and the importance of increasing disease awareness. This study also identified key evidence gaps in Taiwan, highlighting the directions for future research, including the need for developing a unified algorithm to define AA and the severity classification in real-world data sources, as well as to have research to investigate other risk factors and comorbidities with AA. Furthermore, with the publication of a local consensus (68) and the introduction of novel treatment options, disease awareness and diagnosis rates are expected to increase, leading to a more comprehensive understanding of AA epidemiology and disease burden worldwide, ultimately improving patient care and treatment strategies.

## Data Availability

The original contributions presented in the study are included in the article/Supplementary material, further inquiries can be directed to the corresponding author.
