# Supplementary material for: Epidemiology and burden of alopecia areata in Taiwan: a systematic review
Source: Front Med (Lausanne). 2026 Jan 12;12:1723424. doi: 10.3389/fmed.2025.1723424 (PMC12833698; doi:10.3389/fmed.2025.1723424)
Supplement: Supplementary file 2 [file Table_2.docx]

Supplementary Material 2

# Supplementary Tables

Table S1. PICOS search strategy

Table S2. Search strings used in the study and results

Table S3. Quality assessment of included studies using the Joanna Briggs Institute’s critical appraisal checklist by study design: (a) longitudinal analyses, (b) case-control studies, and (c) cohort studies

**Table S1. PICOS search strategy**

| **Criteria** | **Inclusion criteria** |
| --- | --- |
| Population | Alopecia Areata |
| Intervention/Exposure | N/A |
| Comparators | N/A |
| Outcomes | **Epidemiology:** Incidence; prevalence in Taiwan  **Disease burden-**   - **Risk factors:** Diseases or exposures that increased the risk of AA - **Comorbidities :** Comorbidities associated with AA |
| Study design | Region: Taiwan  Inclusion: Clinical trial, observational study  Exclusion: in-vitro study, gene-related study, case report, review articles  Time: 1^st^ January 2010 to 14^th^ June 2024 |

**Table S2. Search strings used in the study and results**

| **Database** | **Search strings** | **Results** |
| --- | --- | --- |
| PubMed | **#1: Alopecia Areata OR "Alopecia Areata"[MESH]** | 6,287 |
|  | #2: Taiwan | 314,932 |
|  | #3: #1 AND #2 | 72 |
|  | #4: #1 AND #2  Filter: Published date from 1^st^ January 2010 | **64** |
| Cochrane | **#1 Alopecia Areata** | 831 |
|  | #2 Taiwan | 4,434 |
|  | #4: #1 AND #2 | **3** |

Search date: 2 July 2024

**Table S3. Quality assessment of included studies using the Joanna Briggs Institute’s critical appraisal checklist by study design: (a) longitudinal analyses, (b) case-control studies, and (c) cohort studies**

**(a) Longitudinal analyses**

| **Year** | **Author** | **Q1** | **Q2** | **Q3** | **Q4** | **Q5** | **Q6** | **Q7** | **Q8** | **Q9** | **Q10** | **Risk of bias** |
| --- | --- | --- | --- | --- | --- | --- | --- | --- | --- | --- | --- | --- |
| 2013 | Wu (24) | Yes | Yes | Yes | Yes | Yes | Yes | Yes | Yes | Yes | Unclear | Low |
| 2016 | Weng (27) | Yes | Yes | Yes | Unclear | Yes | Yes | Yes | Yes | Yes | No | Moderate |
| 2022 | Wong (26) | Yes | Yes | Yes | Yes | Yes | Yes | Yes | Unclear | Yes | Yes | Low |
| 2024 | Tsai (10) | Yes | Yes | Yes | Yes | Yes | Yes | Yes | Yes | Yes | Yes | Low |

**The “Joanna Briggs Institute’s critical appraisal checklist for case series studies” was used to assess the longitudinal analyses:**

Q1: Were there clear criteria for inclusion in the case series?

Q2: Was the condition measured in a standard, reliable way for all participants included in the case series?

Q3: Were valid methods used for identification of the condition for all participants included in the case series?

Q4: Did the case series have consecutive inclusion of participants?

Q5: Did the case series have complete inclusion of participants?

Q6: Was there clear reporting of the demographics of the participants in the study?

Q7: Was there clear reporting of clinical information of the participants?

Q8: Were the outcomes or follow up results of cases clearly reported?

Q9: Was there clear reporting of the presenting site(s)/clinic(s) demographic information?

Q10: Was statistical analysis appropriate?

**(b) Case-control studies**

| **Year** | **Author** | **Q1** | **Q2** | **Q3** | **Q4** | **Q5** | **Q6** | **Q7** | **Q8** | **Q9** | **Q10** | **Risk of bias** |
| --- | --- | --- | --- | --- | --- | --- | --- | --- | --- | --- | --- | --- |
| 2012 | Chu (16) | Yes | Yes | Yes | Yes | Yes | Yes | Yes | Yes | Yes | Yes | Low |
| 2015 | Chung (30) | Yes | Yes | Yes | Yes | Yes | Yes | Yes | Yes | Yes | Yes | Low |
| 2017 | Chiu (69) | Yes | Yes | Yes | Yes | Yes | Yes | Yes | Yes | Yes | Yes | Low |
| 2020 | Li (42) | Yes | Yes | Yes | Yes | Yes | Yes | Yes | Yes | Yes | Yes | Low |
| 2023 | Wang (43) | Yes | Yes | Yes | Yes | Yes | Yes | No^†^ | Yes | Yes | Unclear | Moderate |

^†^Bias was considered minimal since the baseline characteristics were comparable between groups. In addition, the temporal sequence of events is illustrated in Figure 1.

**The “Joanna Briggs Institute’s critical appraisal checklist for case-control studies” was used:**

Q1: Were the groups comparable other than the presence of disease in cases or the absence of disease in controls?

Q2: Were cases and controls matched appropriately?

Q3: Were the same criteria used for identification of cases and controls?

Q4: Was exposure measured in a standard, valid and reliable way?

Q5: Was exposure measured in the same way for cases and controls?

Q6: Were confounding factors identified?

Q7: Were strategies to deal with confounding factors stated?

Q8: Were outcomes assessed in a standard, valid and reliable way for cases and controls?

Q9: Was the exposure period of interest long enough to be meaningful?

Q10: Was appropriate statistical analysis used?

**(c) Cohort studies**

| **Year** | **Author** | **Q1** | **Q2** | **Q3** | **Q4** | **Q5** | **Q6** | **Q7** | **Q8** | **Q9**^†^ | **Q10**^†^ | **Q11** | **Risk of bias** |
| --- | --- | --- | --- | --- | --- | --- | --- | --- | --- | --- | --- | --- | --- |
| 2011 | Chu (15) | Yes | Yes | Yes | Yes | Yes | Yes | Yes | Yes | Unclear | Unclear | Yes | Low |
| 2011 | Tsai (28) | Yes | Yes | Yes | Yes | Yes | Yes | Yes | Unclear | Unclear | Unclear | Yes | Moderate |
| 2015 | Kang (17) | Yes | Yes | Yes | Yes | Yes | Yes | Yes | Yes | Unclear | Unclear | Yes | Low |
| 2015 | Chen (31) | Yes | Yes | Yes | Yes | Yes | Yes | Yes | Yes | Unclear | Unclear | Yes | Low |
| 2015 | Chen (71) | Yes | Yes | Yes | Yes | Yes | Yes | Yes | Yes | Unclear | Unclear | Yes | Low |
| 2016 | Chen (14) | Yes | Yes | Yes | Yes | Yes | Yes | Yes | Yes | Unclear | Unclear | Yes | Low |
| 2018 | Chen (13) | Yes | Yes | Yes | Yes | Yes | Yes | Yes | Yes | Unclear | Unclear | Yes | Low |
| 2019 | Liu (70) | Yes | Yes | Yes | Yes | Yes | Yes | Yes | Yes | Unclear | Unclear | Yes | Low |
| 2020 | Chang (32) | Yes | Yes | Yes | Yes | Yes | Yes | Yes | Yes | Unclear | Unclear | Yes | Low |
| 2020 | Dai (22) | Yes | Yes | Yes | Yes | Yes | Yes | Yes | Unclear | Unclear | Unclear | Yes | Moderate |
| 2020 | Dai (34) | Yes | Yes | Yes | Yes | Yes | Yes | Yes | Yes | Unclear | Unclear | Yes | Low |
| 2020 | Dai (35) | Yes | Yes | Yes | Yes | Yes | Yes | Yes | Yes | Unclear | Unclear | Yes | Low |
| 2020 | Dai (41) | Yes | Yes | Yes | Yes | Yes | Yes | Yes | Yes | Unclear | Unclear | Yes | Low |
| 2020 | Wei (19) | Yes | Yes | Yes | Yes | Yes | Yes | Yes | Yes | Unclear | Unclear | Yes | Low |
| 2020 | Ma (18) | Yes | Yes | Yes | Yes | Yes | Yes | Yes | Yes | Unclear | Unclear | Yes | Low |
| 2021 | Chang (40) | Yes | Yes | Yes | Yes | Yes | Yes | Yes | Yes | Yes | Yes | Yes | Low |
| 2021 | Dai (20) | Yes | Yes | Yes | Yes | Yes | Yes | Yes | Yes | Unclear | Unclear | Yes | Low |
| 2021 | Dai (23) | Yes | Yes | Yes | Yes | Yes | Yes | Yes | Yes | Unclear | Unclear | Yes | Low |
| 2021 | Dai (37) | Yes | Yes | Yes | Yes | Yes | Yes | Yes | Yes | Unclear | Unclear | Yes | Low |
| 2021 | Ho (36) | Yes | Yes | Yes | Yes | Yes | Yes | Yes | Yes | Unclear | Unclear | Yes | Low |
| 2021 | Li (25) | Yes | Yes | Yes | Yes | Yes | Yes | Yes | Yes | Unclear | Unclear | Yes | Low |
| 2021 | Ma (18) | Yes | Yes | Yes | Yes | Yes | Yes | Yes | Yes | Unclear | Unclear | Yes | Low |
| 2021 | Tu (29) | Yes | Yes | Yes | Yes | Yes | Unclear | Yes | Yes | Unclear | Unclear | Yes | Moderate |
| 2022 | Chou (38) | Yes | Yes | Yes | Yes | Yes | Yes | Yes | Yes | Yes | Yes | Yes | Low |
| 2022 | Dai (21) | Yes | Yes | Yes | Yes | Yes | Yes | Yes | Yes | Unclear | Unclear | Yes | Low |
| 2022 | Hsieh (33) | Yes | Yes | Yes | Yes | Yes | Yes | Yes | Yes | Unclear | Unclear | Yes | Low |
| 2022 | Ting (44) | Yes | Yes | Yes | Yes | Yes | Yes | Yes | Unclear | Unclear | Unclear | Yes | Moderate |
| 2023 | Wang (72) | Yes | Yes | Yes | Yes | Yes | Yes | Yes | Yes | Yes | Yes | Yes | Low |

^†^Bias was considered minimal for Q9 and Q10 because, given the nature of the data source (i.e., NHIRD), most patients were covered by Taiwan’s National Health Insurance system and had longitudinal follow-up until death or the end of the data period (Hsieh CY, Clin Epidemiol. 2019;11:349–358. doi:10.2147/CLEP.S196293).

**The “Joanna Briggs Institute’s critical appraisal checklist for cohort studies” was used:**

Q1: Were the two groups similar and recruited from the same population?

Q2: Were the exposures measured similarly to assign people to both exposed and unexposed groups?

Q3: Was the exposure measured in a valid and reliable way?

Q4: Were confounding factors identified?

Q5: Were strategies to deal with confounding factors stated?

Q6: Were the groups/participants free of the outcome at the start of the study (or at the moment of exposure)?

Q7: Were the outcomes measured in a valid and reliable way?

Q8: Was the follow up time reported and sufficient to be long enough for outcomes to occur?

Q9: Was follow up complete, and if not, were the reasons to loss to follow up described and explored?

Q10: Were strategies to address incomplete follow up utilized?

Q11: Was appropriate statistical analysis used?
